# Venetoclax-Resistant MV4-11 Leukemic Cells Activate PI3K/AKT Pathway for Metabolic Reprogramming and Redox Adaptation for Survival

**DOI:** 10.3390/antiox11030461

**Published:** 2022-02-25

**Authors:** Hind A. Alkhatabi, Samir F. Zohny, Mohammed Razeeth Shait Mohammed, Hani Choudhry, Mohd Rehan, Aamir Ahmad, Farid Ahmed, Mohammad Imran Khan

**Affiliations:** 1 Department of Biochemistry, Faculty of Science, King Abdulaziz University, Jeddah 21589, Saudi Arabia; haalkhatabi@uj.edu.sa (H.A.A.); sfzohny@kau.edu.sa (S.F.Z.); razeeth.new@gmail.com (M.R.S.M.); hchoudhry@kau.edu.sa (H.C.); 2 Department of Biochemistry, College of Science, University of Jeddah, Jeddah 21589, Saudi Arabia; 3 Center of Excellence in Genomic Medicine Research, King Abdulaziz University, Jeddah 21589, Saudi Arabia; fahmed1@kau.edu.sa; 4 Centre of Artificial Intelligence for Precision Medicines, King Abdulaziz University, Jeddah 21589, Saudi Arabia; 5 King Fahd Medical Research Centre, King Abdulaziz University, P.O. Box 80216, Jeddah 21589, Saudi Arabia; mrtahir@kau.edu.sa; 6 Translational Research Institute, Hamad Medical Corporation, Doha 3050, Qatar; aahmad9@hamad.qa; 7 Faculty of Applied Medical Sciences, King Abdulaziz University, Jeddah 21589, Saudi Arabia

**Keywords:** venetoclax, acute myeloid leukemia, MV4-11, venetoclax resistance model, metabolomics, PI3K/AKT pathway, glycolysis, redox metabolism, OXPHOS

## Abstract

Venetoclax (ABT199) is a selective B-cell lymphoma 2 (BCL-2) inhibitor. The US FDA recently approved it to be used in combination with low-dose cytarabine or hypomethylating agents in acute myeloid leukemia (AML) or elderly patients non-eligible for chemotherapy. However, acquiring resistance to venetoclax in AML patients is the primary cause of treatment failure. To understand the molecular mechanisms inherent in the resistance to BCL-2 inhibitors, we generated a venetoclax-resistant cell line model and assessed the consequences of this resistance on its metabolic pathways. Untargeted metabolomics data displayed a notable impact of resistance on the PI3K/AKT pathway, the Warburg effect, glycolysis, the TCA cycle, and redox metabolism. The resistant cells showed increased NADPH and reduced glutathione levels, switching their energy metabolism towards glycolysis. PI3K/AKT pathway inhibition shifted resistant cells towards oxidative phosphorylation (OXPHOS). Our results provide a metabolic map of resistant cells that can be used to design novel metabolic targets to challenge venetoclax resistance in AML.

## 1. Introduction

B-cell lymphoma 2 (BCL-2) and other BCL-2-like family members of proteins such as BCL-XL and MCL-1 are highly expressed in leukemia such as acute myeloid leukemia (AML) and account for treatment resistance to chemotherapeutics [1,2]. Several BH3 mimetics have been developed in the past, which bind and block the BH3-binding hydrophobic groove of the pro-survival members of the BCL-2 family. Venetoclax is a BH3 mimetic developed as a highly specific inhibitor of BCL-2 with very low affinity for BCL-XL, unlike the predecessor ABT263 (navitoclax), which additionally inhibits BCL-XL, leading to dose-dependent induction of thrombocytopenia [3]. The binding of venetoclax to the BH-3 domain of BCL-2 leads to the displacement of the pro-apoptotic protein BIM from BCL-2, and the subsequent activation of cytochrome c-mediated intrinsic apoptosis in tumor cells shows a high expression of this protein [4,5]. Venetoclax is now being used to treat adult chronic lymphocytic leukemia and small lymphocytic lymphoma, and in combination with azacytidine, decitabine, or low-dose AraC to treat AML unsuitable for standard chemotherapy [6,7].

Several molecular mechanisms have been proposed to develop venetoclax resistance in AML. TP53 mutation status is a determining factor in the efficacy of venetoclax in AML, and loss of TP53 function impairs BAX/BAK activation, leading to venetoclax resistance [8,9]. It was reported earlier that *FLT3-ITD* or *PTPN11* mutations might confer primary and secondary resistance to venetoclax by enhancing the expression of BCL-XL and MCL-1 [10]. Furthermore, several studies reported that resistance cells significantly alter the metabolic phenotype, as several extracellular factors contributed to metabolic changes. Receptors such as tyrosine kinases, G protein-coupled receptors (GPCRs), and various growth factors are implicated in the PI3K-Akt-mTOR pathway activity. As the PI3K signaling cascade is activated, phosphorylation of phosphatidylinositol 4,5-bisphosphate (PIP2) generates the second messenger phosphatidylinositol 3,4,5-trisphosphate (PIP3). This facilitates the recruitment of the Ser/Thr kinase Akt (protein kinase B). AKT is an important downstream target of PI3K. In contrast, Akt is cytosolic in unstimulated cells, and the translocation of Akt to the membrane, where PIP3 serves as an anchor, requires the activation of PI3K. PIP3 activates Akt, which then downstream activates mTOR. The PI3K-Akt-mTOR pathway knows to regulate cellular metabolism through different mechanisms. mTOR activation induces hypoxia-inducible factor-1 (HIF1); it promotes the glycolysis pathway, resulting in lactate production [11,12].

Metabolic pathway changes in AML have also been associated with resistance to venetoclax in AML. Changes in glutamine levels that mediate mitochondrial oxidative phosphorylation (OXPHOS) might be implicated in the resistance towards venetoclax in AML cells due to mutations in the *IDH3* genes in these tumors [13]. Mitochondrial metabolism contributes to the regulation of primary cellular energy metabolism and oxidative phosphorylation (OXPHOS). Previous studies illustrated the treatment of resistance cells by targeting mitochondrial metabolism [14,15]. Additionally, the RAS/MAPK pathway has been observed to mediate resistance to venetoclax through MCL-1 upregulation, which increases metabolic pathways such as fatty acid and amino acid metabolism that drive OXPHOS, conferring resistance to venetoclax in AML [16]. Lastly, nicotinamide metabolism was shown to mediate resistance to venetoclax in relapsed AML stem cells due to its ability to drive elevated amino acid metabolism via OXPHOS [17].

The studies mentioned above point towards metabolic rearrangement as one of the major pathways for the survival of AML cancer stem cells during venetoclax treatment. This motivated us to perform the current work using untargeted metabolomics, mainly to identify novel/unidentified metabolites related to specific metabolic pathways crucial for the survival of AML cells, resulting in therapeutic resistance towards venetoclax. Our results show that venetoclax-resistant AML cells accumulate metabolites associated with PI3K/AKT pathway activation, shift resistant AML MV4-11 cells towards glycolysis, and facilitate an increased redox potential, which ultimately favors resistance cell survival.

## 2. Materials and Methods

### 2.1. Cell Culture

MV4-11 cells (ATCC, Manassas, VA, USA) were cultured at the Roswell Park Memorial Institute using RPMI 1640 medium (Gibco Life Technologies, New York, NY, USA) that was supplemented with 4.5 g/L glucose, 2 mM L-glutamine, 10% fetal bovine serum (FBS), and ciprofloxacin (10 µg/mL) (Gibco, Thermo Fisher Scientific, Waltham, MA, USA).

### 2.2. Venetoclax (ABT199)-Resistant Model

The generation of the MV4-11 ABT199 resistant line was performed by culturing MV4-11 cells in increasing concentrations of ABT199 (Selleckchem, Radnor, TX, USA, S8048) starting from 1 nM to 100 nM for eight weeks. The cells were first cultured with an initial sub-toxic dose of 1/10th of the baseline IC50 of the cells until significant resistance was reached and cell viability of more than 90% was maintained by the cells with continuous exposures of ABT199 of up to 100 nM, which was 5-fold the IC50 of the parental MV4-11 cell line. The cells were then plated in methylcellulose-based semi-solid media in ABT199-containing medium at a density of 1000 cells/cm^3^ dish (MethoCult™ H4230; Stemcell Technologies, Inc., Vancouver, BC, Canada). The resistant colony was isolated using a light microscope and then cultured using regular RPMI media. This colony was named ABT199-R.

### 2.3. Cell Viability Analysis

The viability of the cells exposed to ABT199 was assessed using the CellTiter-Blue cell viability assay (Promega, Madison, WI, USA, G8081). The cells were seeded in a 96-well plate at a density of 1 × 10⁴ cells per well, and this was carried out in quadruplicate. ABT199 was subsequently added to each of the wells to complete a total volume of 100 µL/well. The plate was incubated at 37 °C for 48 h, followed by the addition of 20 µL of the cell titer-blue reagent to each well and further incubation for 2 h. Fluorescence was measured at 560/590 nm using a multi-mode microplate reader (Spectramax i3, Molecular Devices, LLC, San Jose, CA, USA).

### 2.4. Apoptosis

A total of 1.5 × 10^5^ cells were plated in a 12-well plate, following a 48 h incubation with ABT199. Cell death by apoptosis was assessed by measurements of phosphatidylserine externalization using the PE-Annexin V (BD Bioscience, Franklin Lakes, NJ, USA) and 7-amino-actinomycin D (BD Biosciences) assays with the BD FACS Aria III flow cytometer.

### 2.5. Global Untargeted Metabolomics Profiling Using LC-MS

#### 2.5.1. Metabolite Extraction

To carry out the metabolite extraction assay, firstly, equal numbers of MV4-11 and ABT199-R cells (~3 × 10^6^ cells) that were determined to be in the exponential growth phase were collected, and only the resistant cells were treated with PKI-402 (Selleckchem, Radnor, TX, USA, S2739), which is a dual pan-PI3K/mTOR inhibitor; we selected the dose based on the IC50 value (120 nM and 240 nM). Metabolite extraction was performed on the MV4-11 cells, ABT199-R cells (resistant cells), and ABT-199R cells treated with PKI-402. The cells were immediately lysed by a glass homogenizer using the ice-cold solvent acetonitrile: methanol: water at a ratio of 2:2:1 *v*/*v*. The lysates were vortexed for 30 s, incubated for 1 h at −20 °C, and then centrifuged for 15 min at 13,000 rpm at 4 °C. The supernatant was injected into the LC-MS/MS system [18,19,20].

#### 2.5.2. HPLC Workflow

The HPLC-based separation was carried out before the assay was performed. Briefly, 10 µL of each metabolite extract was injected into an HPLC column (Hypersail gold column C18 Part No: 25005-104630) at a 0.200 mL/min flow rate. Here, 99.9% methanol in formic acid and 0.1% formic acid (0.1%, *v*/*v*) constituted the mobile phase using a gradient program in which the component of the solution was varied from 5% to 30% for 30 min, 30% to 50% for 10 min, 50% for 10 min, and finally 50% to 95% for 20 min, with a total running time of 70 min at a column temperature of 30 °C [20,21].

#### 2.5.3. Mass Spectrometry

An LTQ XL™ linear ion trap (Thermo Fisher Scientific, Waltham, MA, USA) LC-MS/MS instrument was used to analyze the samples with the following MSn parameters: full scanning mode ranged from 80 to 1000 *m*/*z*; the buffer gas that was used in this case was helium, with nitrogen being used as the sheath gas, with 40 arbitrary units as the flow rate; a capillary temperature of 270 °C was used with a voltage of 4.0 V, and the spray voltage was set at −3.0 kV [18,19,20].

#### 2.5.4. Data Analysis

Data analytics was processed using an open access online tool (XCMS database). Features were identified against metabolites that have been deposited in the HMDB (Human Metabolome Database) repository. Statistical and pathway analyses were performed using Metaboanalyst [22].

#### 2.5.5. Data Processing

Data processing was performed by converting the raw file into an mzXML format using raw converter software. The mzXML files were uploaded to XCMS (https://xcmsonline.scripps.edu) accessed on 10 December 2021 and processed for alignment, RT (retention time) correction, and feature detection. The XCMS parameters were set as follows: feature detection using cent-wave settings *m*/*z* wid = 0.25, min frac = 0.5, minimum peak width = 10 s, ∆ *m*/*z* = 30 ppm, and bw = 10 for chromatogram alignment. After retention time evaluation and alignment, a camera was used to detect isotopic peaks and adducts. At 20 ppm accuracy, the precursor was matched with the METLIN database [18].

#### 2.5.6. Metabolomics Pathway Analysis

Metabolomics pathway analysis was carried out using the web-based R package MetaboAnalyst 3.0 (https://www.metaboanalyst.ca/faces/) accessed on 13 December 2021.

### 2.6. Gene Expression Assay

Briefly, 2 × 10^6^ cells/T75 cm^2^ flask of MV4-11, ABT199-R, and ABT199-R treated with 120 nM PKI-402 were cultured for 48 h. According to the manufacturer’s instructions, total RNA was extracted using a Qiagen RNeasy kit (Qiagen, Hilden, Germany). cDNA was synthesized using a reverse transcriptase kit (Promega, Madison, WI, USA). qRT-PCR was performed using SYBRGreen master mix (Life Technologies, Thermo Fisher Scientific, Waltham, MA, USA). Genes related to glycolysis (Glucose transporter 3 (*GLUT-3*)*,* hexokinase 2 (*HK-2*), Phosphofructokinase (*PFKL*), Enolase 2 (*ENO-2*), Lactate Dehydrogenase A (*LDHA-1*), and Pyruvate Dehydrogenase Kinase 1 (*PDK-1*)) and mitochondrial OXPHOS (NADH dehydrogenase 5 (*MT-ND5*), Mitochondrially Cytochrome B (*MT-CYB*) and Mitochondrially Cytochrome C Oxidase III (*MT-CO3*)) were analyzed using a StepOnePlusTM real-time PCR system (Thermo Fisher Scientific, Waltham, MA, USA). Primer sequences are shown in Table 1.

### 2.7. ROS Assay

ROS assays were carried out as follows: Briefly, 4 × 10^5^ cells of MV4-11, ABT199-R, and ABT199-R treated with 120 nM PKI-402 were incubated for 36 h. The reactive oxygen species (ROS) level was measured in live cells using the CellRox Green Flow Cytometry assay kit (C10492; Invitrogen, Waltham, MA, USA). Before flow cytometry analysis was carried out, the cells were firstly incubated in a culture medium supplemented with 500 nM CELLROX for 1 h at 37 °C and 5% CO_2_, and the samples were immediately analyzed by flow cytometry (Amnis^®^ FlowSight^®^), using 488 nm excitation for CellROX^®^ Green; values are taken based on the green fluorescence intensity.

### 2.8. Determination of GSH Levels

The glutathione assay kit (GRSA; Sigma, St. Louis, MO, USA) was used to detect and measure the relatively reduced glutathione (GSH) concentration in MV4-11, ABT199-R, and ABT199-R treated with 120 nM PKI-402 according to the manufacturer’s instructions. Total cell lysates were harvested and used to determine the amount of GSH in each sample using a kinetic assay. The assay is based on the principle that NADPH reduces oxidized glutathione (GSSG) in the presence of glutathione reductase to form GSH. Then, 5,5′-dithiobis (2-nitrobenzoic acid) (DTNB) reacts with GSH to yield 5-thio-2-nitrobenzoic acid (TNB), whose absorbance is measured at 412 nm (Spectramax i3, Molecular Devices, LLC, San Jose, CA, USA).

### 2.9. Western Blot Analysis

Briefly, 10 × 10^6^/T75 cm^2^ flask of MV4-11 cells, ABT199-R cells, and ABT199-R cells treated with 120 nM PKI-402 were cultured for 24 h. The cells were washed with ice-cold PBS (pH 7.4). The cell pellet was resuspended in ice-cold RIPA buffer with a protease inhibitor cocktail (Protease Inhibitor Cocktail Set III, Calbiochem). Then, 4–12% gradient polyacrylamide gels separated 50 µg of protein, which was transferred to an NC nitrocellulose membrane. Ponceau S staining of the membrane was used to confirm the equal loading of the proteins. Appropriate monoclonal primary antibodies against the proteins of interest were used and detected by chemiluminescence using the C-Digit Blot Scanner (Licor, Lincoln, NE, USA) after incubation with specific secondary antibodies (Anti-rabbit IgG -Cell Signaling Technology 7074). The primary antibodies used to probe each of the proteins of interest were as follows: Akt (pan) (C67E7) rabbit monoclonal antibody 4691, and phospho-Akt (Ser473) (D9E) rabbit monoclonal antibody 4060 (Cell Signaling Technology, Danvers, MA, USA).

### 2.10. Statistical Analysis

Statistical and chemometric analyses were performed by Kruskal–Wallis non-parametric analysis. Datasets were presented as the median and interquartile range. Statistical significance was defined as *p* ≤ 0.05.

## 3. Results

### 3.1. ABT199-R Cells Are Highly Resistant to Venetoclax Treatment

**Figure 1:**
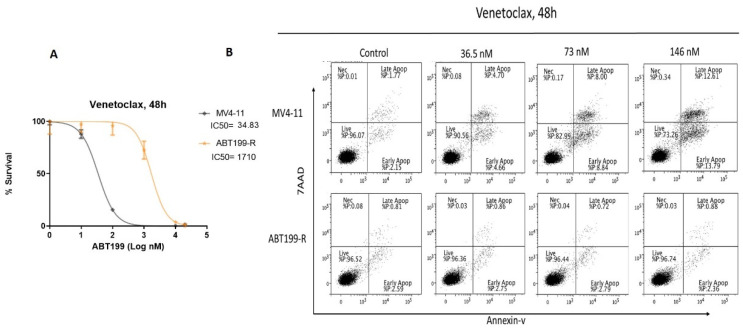
ABT199-R cells are highly resistant to venetoclax. (**A**) MV4-11 and ABT-199R were treated with increasing doses of venetoclax (0–20 µM) for 48 h and assessed with a cell titer blue proliferation assay. The curves indicate the percentage survival of each cell line to increasing doses of venetoclax. (**B**) MV4-11 and ABT199-R cells were treated with indicated concentrations of venetoclax for 48 h and stained for an Annexin-V/7AAD assay. The percentage values determine the early and late apoptotic populations.

We developed the ABT199-R cell line model, as previously mentioned. To validate the resistance of these cells, we evaluated the inhibitory effect of venetoclax on cell viability using a cell titer blue assay. In contrast to the sensitive MV4-11 cells (parental cells), ABT199-R cells showed remarkably higher half-maximal inhibitory concentration (IC50) values in response to venetoclax. ABT199-R cells demonstrated nearly 50-fold higher resistance to venetoclax than the parental cells (Figure 1A).

We further evaluated the cytotoxic effect of ABT199-R with sensitive parental cells (MV4-11). The cells were treated with an increasing concentration above the IC50 for 48 h; resistant cells (ABT199-R) and sensitive cells (MV4-11) were then stained for an Annexin-V/7AAD assay. As revealed in Figure 1B, at a concentration of 36.5, 73, and 146 nM, venetoclax induced apoptotic rates of nearly 10, 20, and 30% amongst the populations of the sensitive cells (MV4-11). In contrast, equal concentrations of venetoclax did not trigger noticeable apoptosis in the resistant cells.

### 3.2. Metabolomics Showed Accumulation of Metabolites Related to PI3K/AKT Pathway in ABT199-R Cells

**Figure 2:**
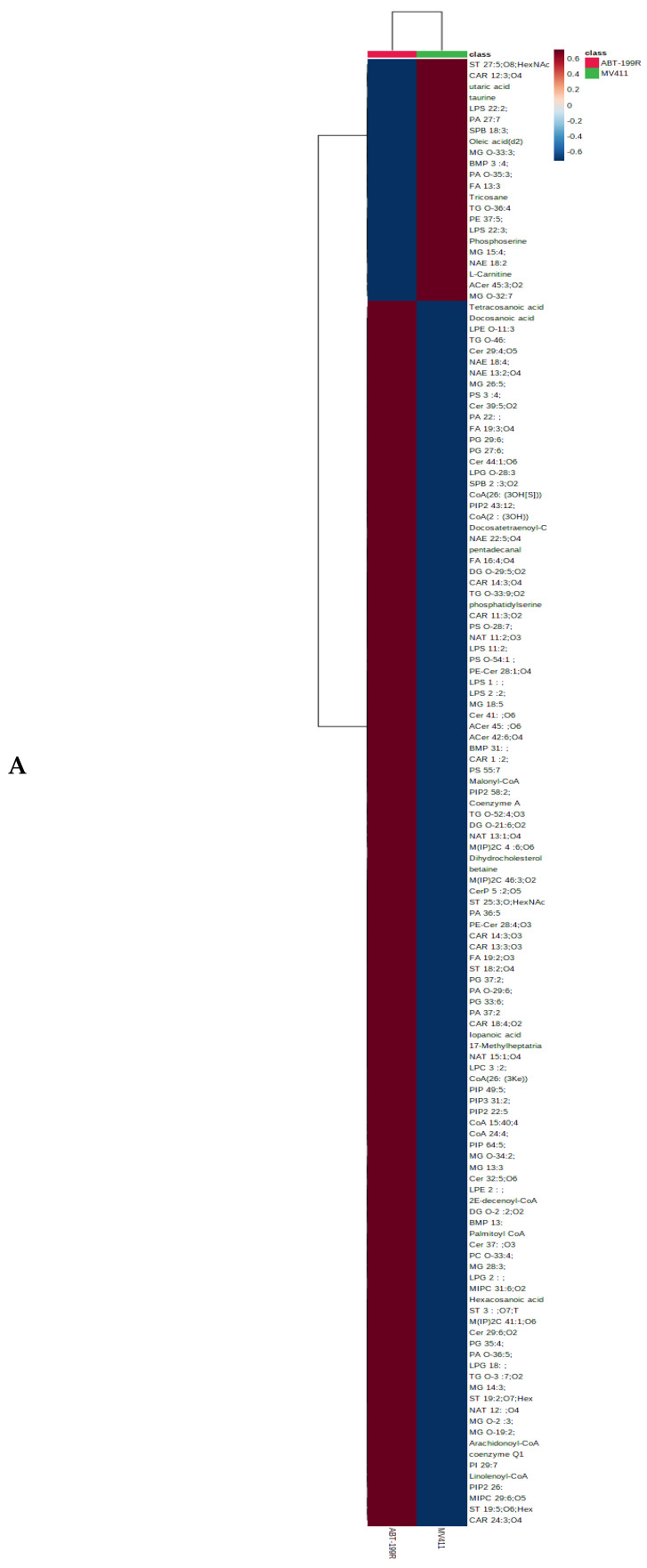

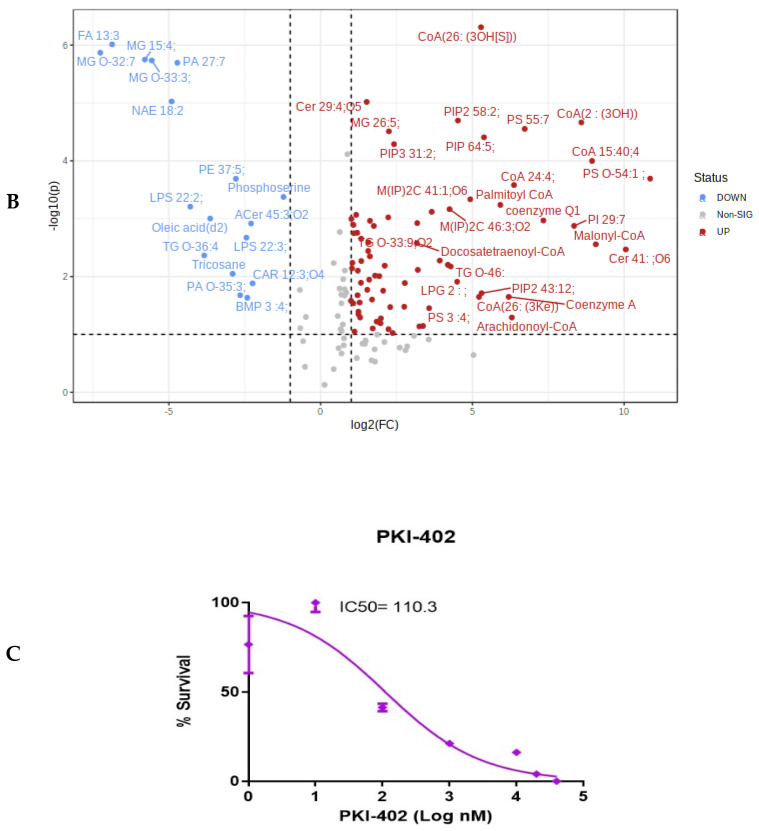
Metabolomic analysis of MV4-11 cells vs. ABT199-R cells. (**A**) The metabolic heatmap profile of differentially accumulated metab olites between sensitive MV4-11 and resistant ABT199-R. (**B**) Volcano plots of metabolic differentiation between MV4-11 and ABT199-R. (**C**) ABT199-R cells were treated with several concentrations of PKI-402 for 48 h and assessed with cell titer blue viability assay. The curve indicates the IC50 value.

To study the metabolic fingerprint of venetoclax-sensitive and resistant cells, we performed untargeted global metabolomics to understand the metabolic variation between MV4-11 and ABT199-R cells. The metabolites’ features were analyzed using the XCMS online database. The evident metabolic variation suggests unique metabolomic profiles for venetoclax-sensitive and resistant cells. The metabolic heatmap and volcano plot demonstrate impairment in the metabolites related to the PIP family, which were highly accumulated in ABT199-R cells compared to the sensitive cells (Figure 2A,B). Therefore, we targeted the PI3K pathway in the resistant cells using the PKI-402 inhibitor. First, we examined the cytotoxicity of PKI-401 on ABT199-R cells to determine the dose of treatment, and the IC50 value was nearly 120 nM (Figure 2C).

### 3.3. Targeting PI3K/AKT Pathway Shifts the Metabolic Profile of Venetoclax-Resistant Cells Closer to MV4-11

**Figure 3:**
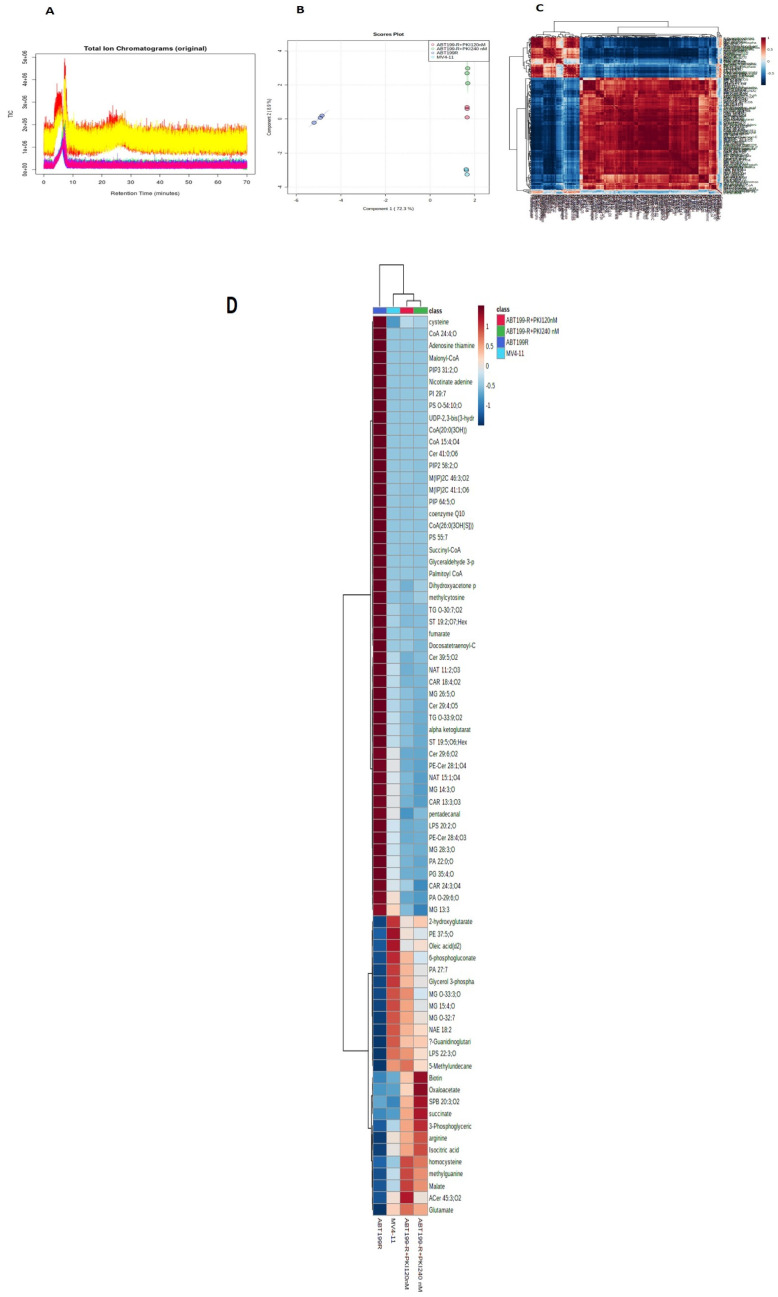

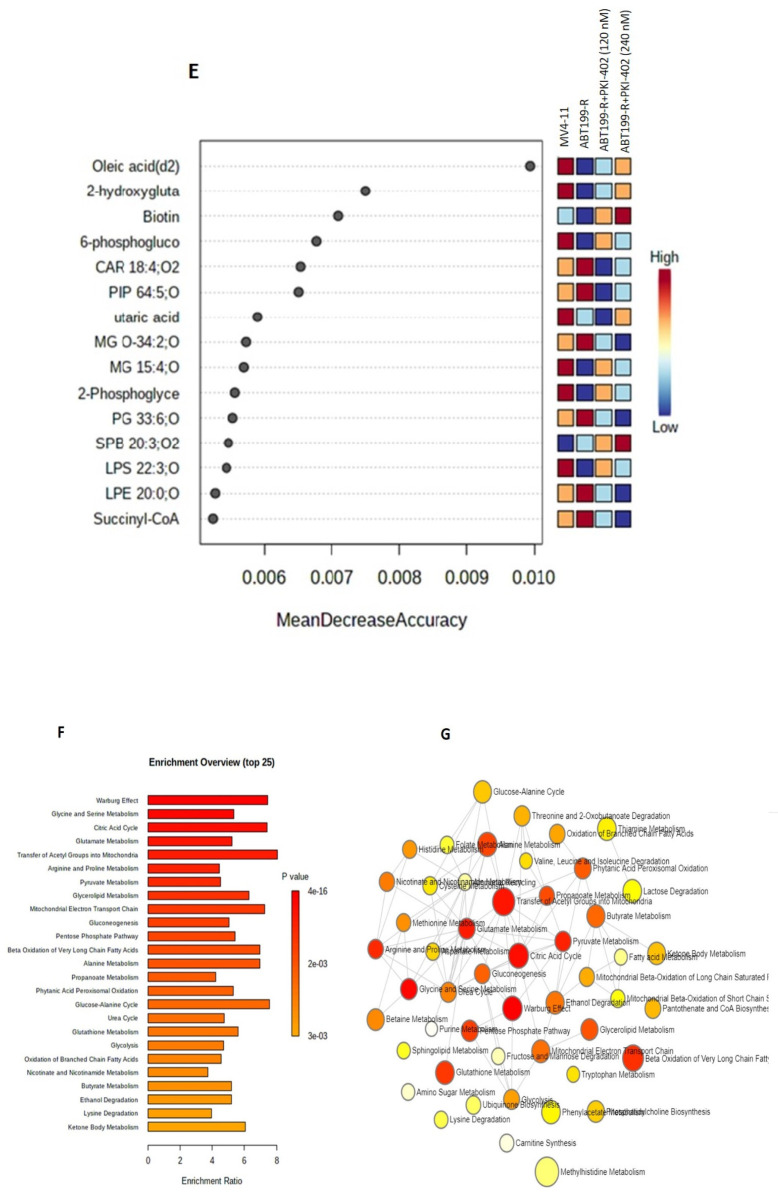
Metabolomic analysis of MV4-11, ABT199-R, and ABT199-R treated with PKI-402. (**A**) Metabolites were extracted and run in LTQ-XL linear ion trap LC-MS, showing their total ion chromatograms. (**B**) PCA analysis of comprehensive metabolites of MV4-11 cells, ABT199-R cells, and ABT199-R cells treated with PKI-402. (**C**) Correlation heatmap of ABT-199R cells, MV4-11 cells, and ABT199-R cells treated with PKI-402. (**D**) Heatmap of differentially expressed metabolites in MV4-11 cells, ABT199-R cells, and ABT199-R cells treated with PKI-402. (**E**) VIP score based on PCA analysis of principal metabolites. (**F**) Top pathway enriched in metabolome analysis in MV4-11 cells, ABT199-R cells, and ABT199-R cells treated with PKI-402. (**G**) Pathway network analysis.

We further evaluated global metabolomics among MV4-11 cells, ABT199-R cells, and ABT199-R cells treated with PKI-402. A total of 513 features were identified using ESI positive mode analysis, and 273 metabolites showed a significant change (*p* ≤ 0.05) (Figure 3A).

Principal component analysis (PCA) indicated a clear difference in metabolomics among the MV4-11 cells, ABT199-R cells, and ABT199-R cells treated with PKI-402 (Figure 3B); the correlation coefficient heatmap showed self-correlations between metabolites (Figure 3C). The heatmap for the metabolites demonstrated the difference in metabolic accumulation among the MV4-11 cells, ABT199-R cells, and ABT199-R cells treated with PKI-402 (Figure 3D). This result shows that PKI-402 treatment, specifically 120 nM, shifts the metabolic phenotype of ABT199-R cells closer to MV4-11 cells.

The highly significant metabolite with its expression index in PCA analysis revealed the change in metabolite accumulation in MV4-11 cells, ABT199-R cells, and ABT199-R cells treated with PKI-402 (Figure 3E). The top 181 metabolites with their peak intensity values are presented in Supplementary Table S1, and *p*-values with the raw data are provided in Supplementary Table S2. The pathway analysis and pathway linkage analysis of ABT199-R cells and ABT199-R cells treated with PKI-402 compared with MV4-11 cells were acquired through MetaboAnalyst 5.0. A total of 94 metabolic pathways were enriched. The top 25 metabolic enriched pathways are shown in Figure 3F, mainly involved in the Warburg effect, glycine and serine metabolism, the mitochondrial electron transport chain, glycolysis, gluconeogenesis, the pentose phosphate pathway, glutamate, glutathione metabolism, the TCA cycle, and β-oxidation for long-chain fatty acid and some other metabolic pathways such as one-carbon metabolism, fatty acid pathways, and nicotinamide metabolism (Figure 3F). Pathway linkage analysis showed that metabolites are interlinked with multiple pathways (Figure 3G).

### 3.4. ABT-199R Cells Maintain Heightened p-AKT Signaling

**Figure 4:**
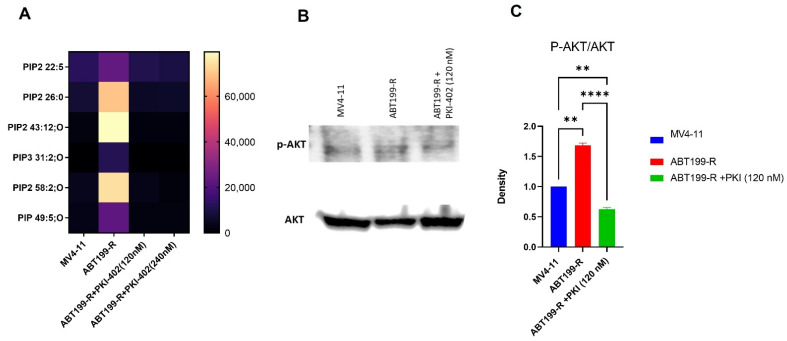
p-AKT signaling pathway in ABT199-R cells. (**A**) Expression of PIP metabolites in MV4-11 cells, ABT199-R cells, and ABT199-R cells treated with PKI-402. (**B**) Western blot analysis of p-AKT and AKT in MV4-11 cells, ABT199-R cells, and ABT199-R cells treated with PKI-402. (**C**) Densitometer analysis of Western blot analysis of p-AKT/AKT in MV4-11 cells, ABT199-R cells, and ABT199-R cells treated with PKI-402. ** *p* < 0.01 and **** *p* < 0.0001.

p-AKT (AKT serine/threonine kinase 1), also referred to as protein kinase B (PKB), is an essential target of phosphatidylinositol 3-kinase (PI3K). Since PIP secondary metabolites activate the PI3K-AKT-mTOR pathway, targeting this pathway may reduce PIP secondary metabolites and AKT phosphorylation. Our results show that PKI-402 treatment significantly reduced PIP secondary metabolites (Figure 4A) and further reduced AKT phosphorylation (Figure 4B,C).

### 3.5. ABT199-R Cells Possess High Glycolytic Phenotypes

**Figure 5:**
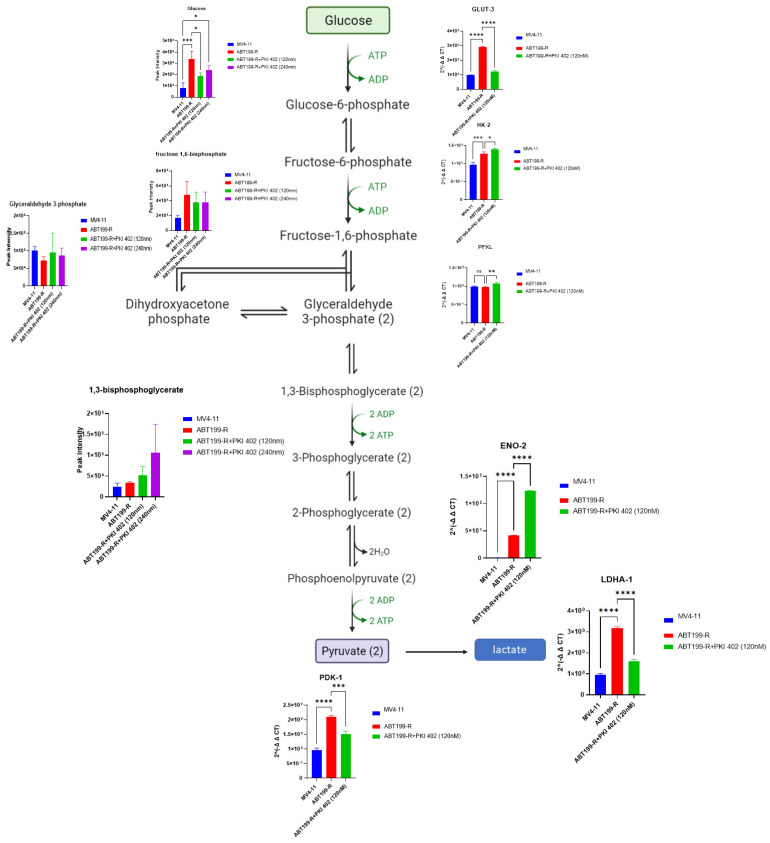
Venetoclax-resistant ABT199-R cells alter the glycolysis pathway. (**Left**) Quantitative levels of various metabolites involved in glycolysis pathways of MV4-11 cells, ABT199-R cells, and ABT199-R cells treated with PKI-402. (**Right**) Gene expression (RT-PCR) of various genes involved in glycolysis. * *p* < 0.05, ** *p* < 0.01, *** *p* < 0.001, and **** *p* < 0.0001.

Venetoclax-resistant cells revealed pathways involved in multiple cellular metabolisms. To determine whether venetoclax resistance is linked to glycolysis, we first measured the glycolytic intermediates in venetoclax-resistant cells (ABT199-R). Indeed, we observed higher glycolytic metabolites in venetoclax-resistant cells (ABT199-R) in comparison to their parental cells (MV4-11) (Figure 5). These results indicate a glycolytic shift in venetoclax-resistant cells.

We next assessed whether key glycolytic network gene transcripts were changed upon venetoclax resistance and in venetoclax-resistant cells treated with PKI-402. Interestingly, the expression of *GLUT-3*, *LDHA1*, and *PDK1* was significantly upregulated in venetoclax-resistant ABT199-R cells and downregulated upon PKI-402 treatment compared to the parental MV4-11 cells (Figure 5).

### 3.6. ABT199-R Cells Exhibit Decrease in TCA Cycle and OXPHOS Metabolites

**Figure 6:**
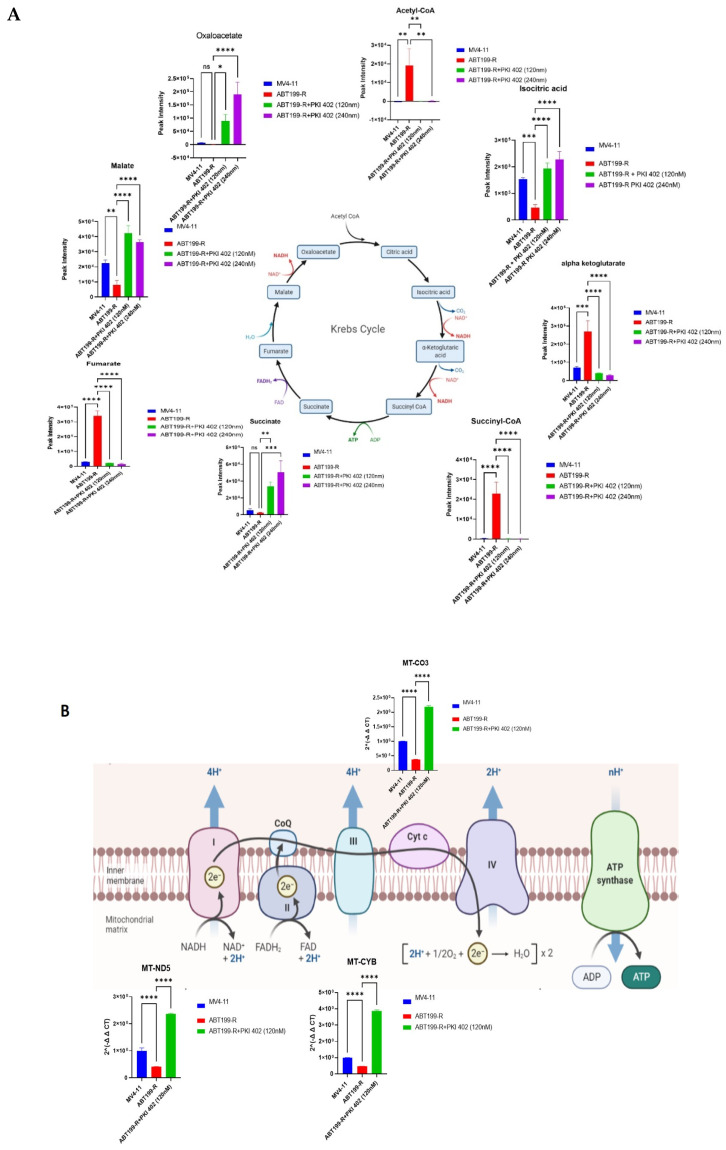
TCA cycle metabolism and OXPHOS are altered in ABT199-R cells. (**A**) The peak intensity of individual metabolites and their quantitative levels involved in the TCA cycle of MV4-11 cells, ABT199-R cells, and ABT199-R cells treated with PKI-402. (**B**) Gene expression (RT-PCR) of various genes involved in mitochondrial OXPHOS. ns = non-significant, * *p* < 0.05, ** *p* < 0.01, *** *p* < 0.001, and **** *p* < 0.0001.

We examined the mitochondrial OXPHOS of venetoclax-resistant cells. The intermediate metabolites of the TCA cycle in ABT199-R cells were reduced to a greater extent than in MV4-11 cells, such as malate, glutamate, oxaloacetate, succinate, 2-hydroxy glutamate, and isocitric acid (Figure 6A). However, PKI-402 treatment increased TCA cycle metabolites in ABT199-R (Figure 5). Consistent with these results, mitochondrial OXPHOS genes such as *MT-ND5*, *MT-CO3*, and *MT-CYB* were also downregulated in ABT199-R cells and upregulated in PKI-402-treated cells (Figure 6B), suggesting that resistant cells treated with PKI-402 shift the metabolism to OXPHOS.

### 3.7. ABT199-R Cells Possess High Redox Potential

**Figure 7:**
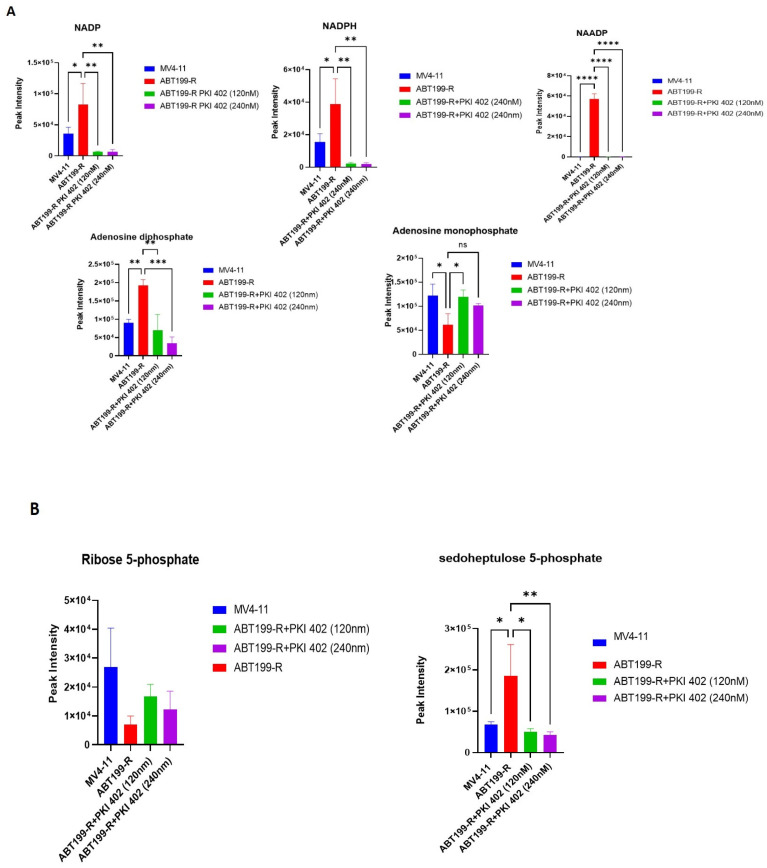
Pentose phosphate pathway (PPP) and energy metabolites modified in ABT199-R cells. (**A**) The density of metabolic features involved in regulating the energy metabolism of MV4-11 cells, ABT199-R cells, and ABT199-R cells treated with PKI-402. (**B**) Metabolites involved in PPP. ns = non-significant, * *p* < 0.05, ** *p* < 0.01, *** *p* < 0.001, and **** *p* < 0.0001.

**Figure 8:**
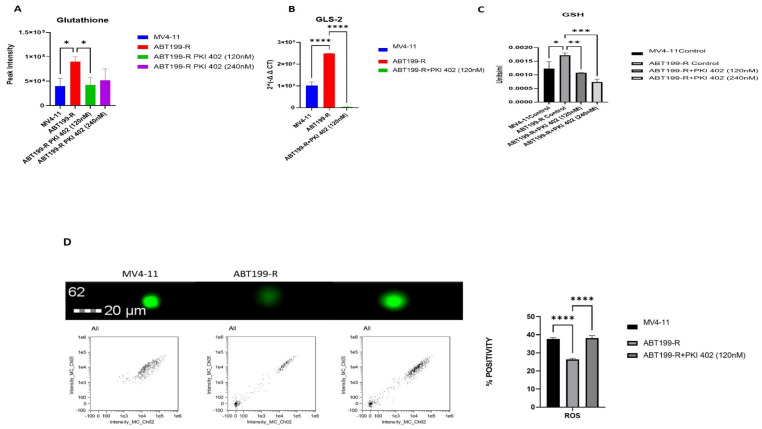
Redox homeostasis of venetoclax-resistant cells. (**A**) Glutathione (metabolite) involved in GSH pathway. (**B**) GLS-2 (gene) is involved in GSH pathway expression (RT-PCR). (**C**) Relative glutathione concentrations were measured using GSH assay from total protein. (**D**) ROS levels using CellRox Green Flow Cytometry assay. The channel Ch05 scans for red, and Ch02 scans for green. * *p* < 0.05, ** *p* < 0.01, *** *p* < 0.001, and **** *p* < 0.0001.

NADPH plays a crucial role in maintaining cellular antioxidation systems, and it is used as a cofactor by glutathione reductase to reduce GSSG into GSH. We observed that NADPH, NADP, and NAADP increased in resistant ABT199-R cells (Figure 7A). We further assessed the intermediate metabolites of the pentose phosphate pathway (PPP), as PPP is a major pathway that generates NADPH. Certainly, sedoheptulose-5-phosphate was significantly increased in ABT199-R cells and decreased upon treatment with PKI-402 (Figure 7B). Moreover, GSH is a critical non-enzymatic ROS antioxidant related to other enzymes that participate in free radical detoxification. Interestingly, the level of GSH is higher in the resistant ABT199-R cells, while ROS levels decreased compared to the sensitive parental MV4-11 cells. PKI-402 treatment reversed glutathione metabolism and induced ROS levels in ABT199-R (Figure 8A–D).

### 3.8. PI3K/AKT Pathway Inhibition Promotes Apoptosis in ABT199-R Cells

**Figure 9:**
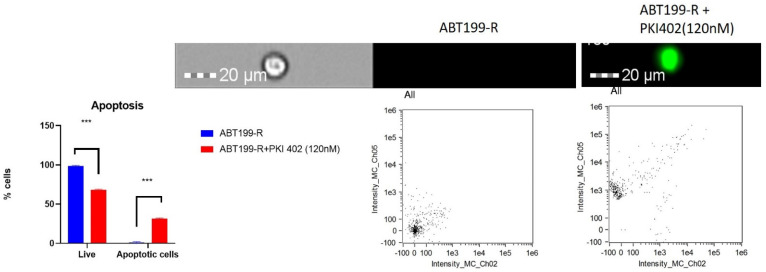
Inhibition of PI3K-AKT induces apoptosis in resistant ABT199-R cells: ABT199-R cells were treated with the IC50 value of PKI-402 for 48 h and stained for an Annexin-V/PI assay. The single-cell images were captured using Amnis^®^ FlowSight^®^. The percentage of apoptosis was calculated based on cell positivity. The channel Ch05 scans for red, and Ch02 scans for green. *** *p* < 0.001.

PKI-402 treatment inhibited cell proliferation in ABT199-R cells. Annexin V/PI staining was used to stain apoptotic cells. The overall apoptotic rate was calculated as the apoptotic rate of cells in channel 2. We observed that PKI-402 treatment induced 30% apoptosis in ABT199-R (Figure 9).

## 4. Discussion

Venetoclax is a clinically approved BH3 mimetic that induces apoptosis in cells by selectively targeting BCL-2. Even though venetoclax shows promising early clinical outcomes in AML, acquired resistance to venetoclax is a significant challenge in its successful use. A recent study reported that venetoclax has metabolic effects independent of its BCL-2 inhibitory function [13]. Our goal was to characterize the metabolic features of venetoclax-resistant cells and compare them to the non-resistant parental cells. We used global untargeted metabolomics to identify crucial pathways mediating venetoclax resistance. We observed that the sensitive MV4-11 and the resistant ABT199-R cells showed different outlines of metabolism based on metabolomic data. Integrated pathway analysis revealed that altered metabolic networks in the resistant cells were related to carbon energy metabolism, including the Warburg effect, glycolysis, the pentose phosphate pathway, glutamate, and the TCA cycle.

The production of PIP3 activates the PI3K/AKT pathway. It is an essential signaling pathway modulating multidrug resistance (MDR) in various types of cancer, such as leukemia, breast cancer, ovarian cancer, lung cancer, hepatocellular carcinoma, and melanoma [23,24,25,26,27,28]. The drug resistance phenotype is often associated with activation of the PI3K/AKT pathway, which provides a survival signal against cytotoxic anticancer drugs and enhances the cancer stem cell (CSC) phenotype. Our data show the accumulation of the PIP family’s metabolites in ABT199-R cells. PI3K/AKT signaling regulates insulin receptors and controls glucose uptake and glycogen synthesis [24]. We observed that glycolysis was enhanced in resistant cells, and glucose transporter GLUT-3 expression increased. Additionally, our data show a reduction in enzymes involved in mitochondrial OXPHOS that leads to a shift in energy metabolism towards glycolysis. The inhibition of the PI3K/AKT/mTOR signaling pathway in ABT199-R using PIK-402 reduced the phosphorylated forms of phosphatidylinositol (phosphoinositides) (PIP). Subsequently, the expression of GLUT-3 and glycolytic intermediates was reduced.

NADPH is a crucial antioxidant and essential for the regeneration of GSH to uphold redox homeostasis [26]. The stability between ROS elimination and generation governs the cellular redox state. A previous study reported that venetoclax-resistant stem cells exhibited a higher level of NAD^+^, a finding that was corroborated in the present study [17]. We hypothesized that the elevated NADPH regulates redox homeostasis. Our data demonstrate that venetoclax-resistant cells have higher NADPH levels, and the metabolites integrated into the maintenance of redox homeostasis were altered in resistant cells (ABT199-R). NADPH, NADP, NAADP, and GSH were elevated in resistant cells compared to the sensitive parental cells. However, venetoclax-resistant cells maintain a high level of NADPH and GSH. NADPH metabolism is co-related to mitochondrial metabolism and ROS generation. Normal mitochondrial function maintains cancer cell survival and proliferation. Metabolites involved in mitochondrial metabolism such as pyruvate regulate the mitochondrial polarization and generation of mitochondrial ROS. Mitochondrial metabolism plays a crucial role in driving multiple cellular functions, and mitochondria-derived ROS have an important effect on pluripotency gene expression that drives the reprogramming of cancer cells [29]. Mitochondrial ROS induce redox-sensitive transcription factors via GSH and AKT signaling. Suppression of mitochondrial metabolism attenuates ROS generation, while the high concentration of ROS acts as an inhibitory effect on cell proliferation [30]. This is most likely responsible for the reduced ROS production observed in resistant cells due to the downregulation of OXPHOS and thereby for reducing mitochondrial ROS generation. Our study demonstrates a reduction in the GSH level, and the induction of OXPHOS and cellular ROS, by targeting the PI3K-AKT pathway.

## 5. Conclusions

In this study, we revealed that venetoclax-resistant cells modulate intermediatory cellular metabolism and antioxidant levels. Further, venetoclax-resistant cells showed increased NADPH, which leads to increased overall energy metabolism. Higher GSH and lower ROS levels were observed in venetoclax-resistant cells. Inhibition of the PI3K/AKT pathway in ABT199-R changed the energy metabolism towards OXPHOS and caused a reduction in GSH levels. Furthermore, the elevated ROS level in PKI-402-treated resistant cells was shown to induce apoptosis. We conclude that venetoclax resistance is associated with activation of the PI3K/AKT pathway, and that treating the resistant cell line with a PIK-402 inhibitor shifts metabolism from glycolysis to OXPHOS. Our data provide the identification of novel metabolic therapeutic targets in venetoclax-resistant MV4-11 cells in AML.

## Figures and Tables

**Table 1:** Genes and primer sequences used for quantitative RT-PCR.

Gene ID	Genes	Primers
6515	*GLUT-3*	F: 5′-TGCCTTTGGCACTCTCAACCAG-3′ R: 5′-GCCATAGCTCTTCAGACCCAAG-3′
3099	*HK-2*	F: 5′-GAGTTTGACCTGGATGTGGTTGC-3′ R: 5′-CCTCCATGTAGCAGGCATTGCT-3′
5211	*PFKL*	F: 5′-AAGAAGTAGGCTGGCACGACGT-3′ R: 5′-GCGGATGTTCTCCACAATGGAC-3′
2026	*ENO-2*	F: 5′-TCATGGTGAGTCATCGCTCAGGAG-3′ R: 5′-ATGTCCGGCAAAGCGAGCTTCATC-3′
3939	*LDHA*	F: 5′-GGATCTCCAACATGGCAGCCTT-3′ R: 5′-AGACGGCTTTCTCCCTCTTGCT-3′
5163	*PDK-1*	F: 5′-CATGTCACGCTGGGTAATGAGG-3′ R: 5′-CTCAACACGAGGTCTTGGTGCA-3′
4540	*MT-ND5*	F: 5′-TCACTTCAACCTCCCTCACC-3′ R: 5′-CAGGGAGGTAGCGATGAGAG-3′
4519	*MT-CYB*	F: 5′-ATCACTCGAGACGTAAATTATGGCT-3′ R: 5′-TGAACTAGGTCTGTCCCAATGTATG-3′
4514	*MT-CO3*	F: 5′-AGTAAGCCTCTACCTGCACG-3′ R: 5′-GAGGAGCGTTATGGAGTGGA-3′
6175	*RPLP0*	F: 5′-TGGTCATCCAGCAGGTGTTCGA-3′ R: 5′-ACAGACACTGGCAACATTGCGG-3′

## Data Availability

The data presented in this study are available on request from the corresponding author.

## Supplementary Materials

The following supporting information can be downloaded at: https://www.mdpi.com/article/10.3390/antiox11030461/s1, Table S1: The peak intensity of significant metabolites; Table S2: Raw data analyzed after XCMS.
